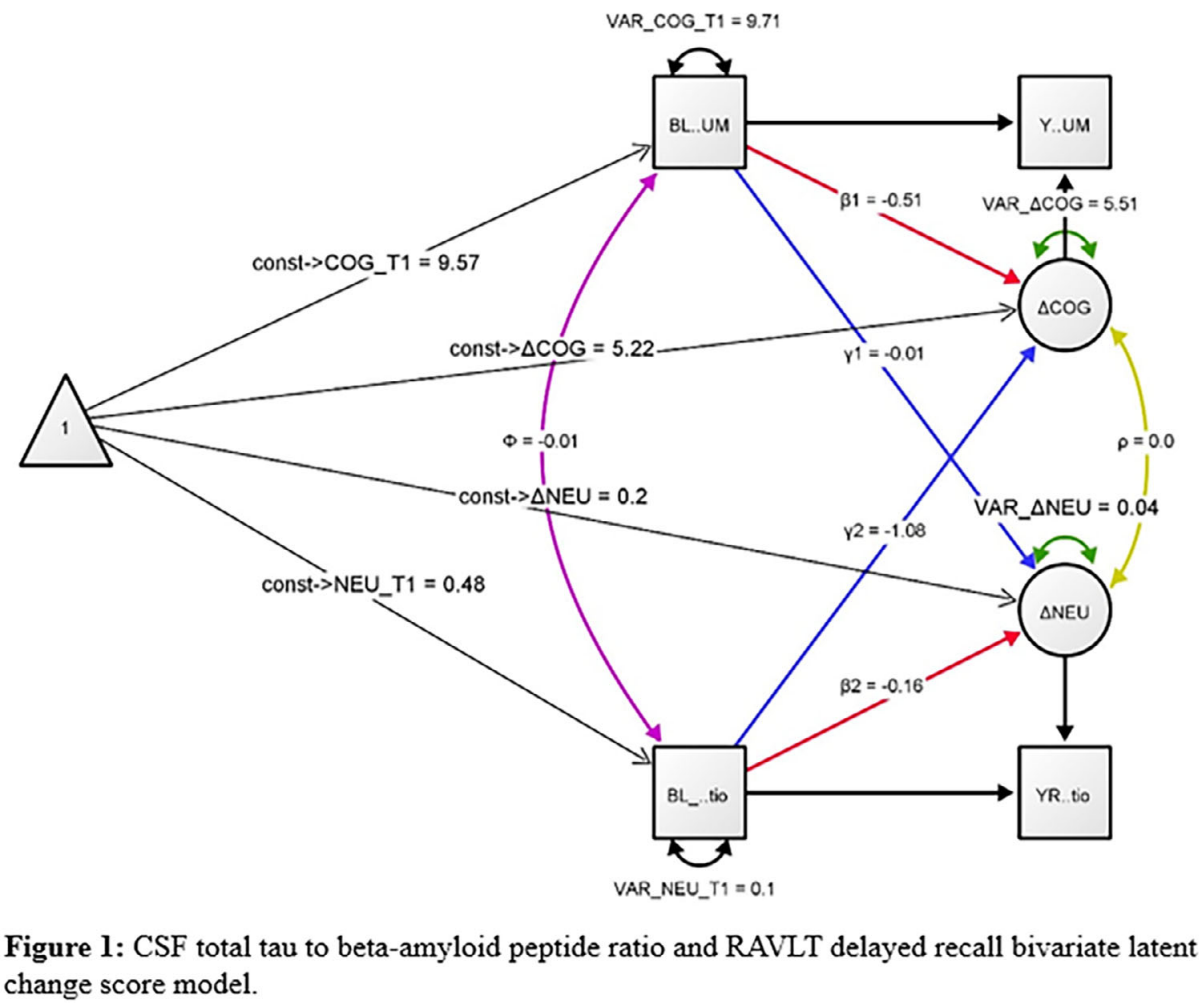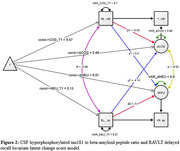# Delayed recall predicts change in cerebrospinal fluid biomarkers over two years

**DOI:** 10.1002/alz70857_106534

**Published:** 2025-12-26

**Authors:** Bradley J. Dixon, John L. Woodard, Lynn M Bekris, Maria Khrestian, Frank P. DiFilippo, Kristy A. Nielson, J. Carson Smith, Sally Durgerian, Stephen M. Rao

**Affiliations:** ^1^ Wayne State University, Detroit, MI, USA; ^2^ Cleveland Clinic, Cleveland, OH, USA; ^3^ Marquette University, Milwaukee, WI, USA; ^4^ University of Maryland, College Park, MD, USA

## Abstract

**Background:**

Performance on neuropsychological measures can be used to predict conversion to Alzheimer's disease. However, few studies have examined the relationship between neuropsychological test performance and the presence of biomarkers (Gainotti et al., 2014). Neuropsychological approaches that are sensitive enough to detect subtle cognitive changes specific to Alzheimer's disease pathology before functional declines are observed will be essential for early identification and effective intervention. van Harten et al. (2021) found that cerebrospinal fluid (CSF) total tau (t‐tau) to beta‐amyloid peptide (Aβ_1‐42_) and hyperphosphorylated tau181 (*p*‐tau181) to Aβ_1‐42_ ratios are robust indicators of Alzheimer's disease. The present study hypothesized that a change in neuropsychological test performance would predict a change in t‐tau/Aβ_1‐42_ and *p*‐tau181/Aβ_1‐42_ ratios.

**Method:**

Participants included 137 healthy older adults (52% female) who underwent baseline neuropsychological testing and lumbar puncture. Participants completed the Rey Auditory Verbal Learning Test (RAVLT), and CSF t‐tau, *p*‐tau181, and Aβ_1‐42_ were collected via lumbar puncture as part of a more extensive evaluation. Follow‐up testing was completed two years later with 92 participants. A bivariate latent change score model was used to analyze the longitudinal relationships between baseline and latent two‐year change for delayed recall on the RAVLT with the CSF t‐tau/Aβ_1‐42_ and *p*‐tau181/Aβ_1‐42_ ratios.

**Result:**

Baseline delayed recall scores were negatively associated with a two‐year change in the CSF t‐tau/Aβ_1‐42_ ratio (γ1 = ‐0.01, *z* = 0.0068, *p* = 0.036). Baseline CSF *p*‐tau181/Aβ_1‐42_ ratio was negatively associated with a two‐year change in delayed recall scores (γ2 = ‐4.14, *z* = ‐2.01, *p* = 0.046). These results suggest that delayed recall on a verbal learning task can predict a change in the CSF t‐tau/Aβ_1‐42_ ratio and that CSF *p*‐tau181/Aβ_1‐42_ ratios can predict change in delayed recall among cognitively healthy older adults.

**Conclusion:**

These findings support the hypothesis that neuropsychological test performance, specifically delayed recall, is sensitive to a two‐year change in some Alzheimer's disease biomarkers while other biomarkers predict change in neuropsychological test performance. Therefore, delayed recall tasks may be practical screening tests for early intervention studies to identify persons at high risk for AD while other biomarkers can predict decline in memory.